# Comparative Evaluation of the Short-Term Outcome of Different Endovascular Aortic Arch Procedures

**DOI:** 10.3390/jcm13164594

**Published:** 2024-08-06

**Authors:** Artis Knapsis, Melik-Murathan Seker, Hubert Schelzig, Markus U. Wagenhäuser

**Affiliations:** Department of Vascular and Endovascular Surgery, Medical Faculty, University Hospital Duesseldorf, Heinrich-Heine-University, 40225 Duesseldorf, Germany; melik-murathan.seker@uni-duesseldorf.de (M.-M.S.); hubert.schelzig@med.uni-duesseldorf.de (H.S.); markus.wagenhaeuser@med.uni-duesseldorf.de (M.U.W.)

**Keywords:** thoracic endovascular aortic repair, fenestrated thoracic stent graft, branched thoracic stent graft, custom-made thoracic stent graft, in situ fenestration, chimney technique

## Abstract

**Objectives**: There are several endovascular treatment options to treat aortic arch and thoracic aortic pathologies with custom-made or surgeon-modified aortic stent grafts. This study seeks to assess endovascular treatment methods for aortic arch and thoracic aortic pathologies with no acceptable proximal landing zone for standard thoracic endovascular aortic repair (TEVAR), comparing different treatment methods and evaluating technical success, intraoperative parameters and short-term outcomes. **Methods**: All patients undergoing elective or emergency endovascular treatment of aortic arch and thoracic aortic pathologies, with no acceptable landing zone for standard TEVAR, between 1 January 2010 and 31 March 2024, at the University Hospital Düsseldorf, Germany were included. An acceptable landing zone was defined as a minimum of 2 cm for sufficient sealing. All patients were not suitable for open surgery. Patients were categorized by an endovascular treatment method for a comprehensive comparison of pre-, intra- and postoperative variables. IBM SPSS29 was used for data analysis. **Results**: The patient cohort comprised 21 patients, predominantly males (81%), with an average age of 70.9 ± 9 years with no acceptable proximal landing zone for standard TEVAR procedure. The most treated aortic pathologies were penetrating aortic ulcers and chronic post-dissection aneurysms. Patients were sub-grouped according to the applied procedure as follows: five patients with chimney thoracic endovascular aortic repair (chTEVAR), seven patients with in situ fenestrated thoracic endovascular aortic repair (isfTEVAR), six patients with custom-made fenestrated thoracic endovascular aortic repair (cmfTEVAR) and three patients with custom-made branched thoracic endovascular aortic repair (cmbTEVAR). Emergency procedures involved two patients. There were significant differences in the total procedure and fluoroscopy time, as well as in contrast agent usage among the treatment groups. cmfTEVAR had the shortest total procedure time, while chTEVAR exhibited the highest contrast agent usage. The overall mortality rate among all procedures was 9.5% (two patients) and 4.7% for elective procedures, respectively. Deaths were associated with either retrograde type A dissection or stent graft infection. Both patients were treated with chTEVAR. There was one minor and one major stroke; these patients were treated with isfTEVAR. No endoleak occurred during any procedure. The reintervention rate for chTEVAR was 20% and 0% for all other procedures during the in-hospital stay. The patients who were treated with cmfTEVAR had no complications, the shortest operating and fluoroscopy time, and less contrast agent was needed in comparison with other treatment methods. **Conclusions**: Complex endovascular procedures of the aortic arch with custom-made or surgeon-modified aortic stent grafts offer a safe solution, with acceptable complication rates for patients who are not suitable for open aortic arch repair. In terms of procedure-related parameters and complication rates, a custom-made fenestrated TEVAR is potentially advantageous compared to the other endovascular techniques.

## 1. Introduction

Pathologies of the aortic arch can be treated either through open surgery, hybrid approaches with debranching of supra-aortic vessels or endovascular methods with custom-made or surgeon-modified fenestrated or branched aortic stent grafts. Aortic arch debranching and TEVAR for type B aortic dissections are associated with significant mortality [1].

One of the crucial advantages of endovascular therapy is the minimally invasive access, resulting in lower stress on the cardiovascular system and consequently reduced perioperative morbidity and mortality. However, not every anatomy is suitable for a conventional thoracic endovascular aortic repair (TEVAR), often lacking the proximal or distal landing zone for a secure implantation. Additionally, patients often present with advanced age and frequently have numerous comorbidities. For this patient cohort, newer endovascular procedures exist, involving patient-specific custom-made fenestrated (cmfTEVAR) or branched thoracic endovascular aortic grafts (cmbTEVAR) for the treatment of aortic arch pathologies. These procedures are extended by the possibility of in situ fenestration (isfTEVAR) or chimney TEVAR (chTEVAR) in case urgent treatment is warranted.

ChTEVAR describes a procedure in which the supra-aortic branches are supplied with stent grafts alongside the primary aortic stent graft, mostly in emergency settings. When these stent grafts are deployed in the side branches parallel to the aortic stent graft, a proximal landing zone can be created, maintaining continuous perfusion of the aortic side branches. The goal is a treatment of pathologies regarding Zones 2 and 3 (based on Ishimaru zones) and the following zones, depending on the severity of the disease [2].

**Figure 1:**
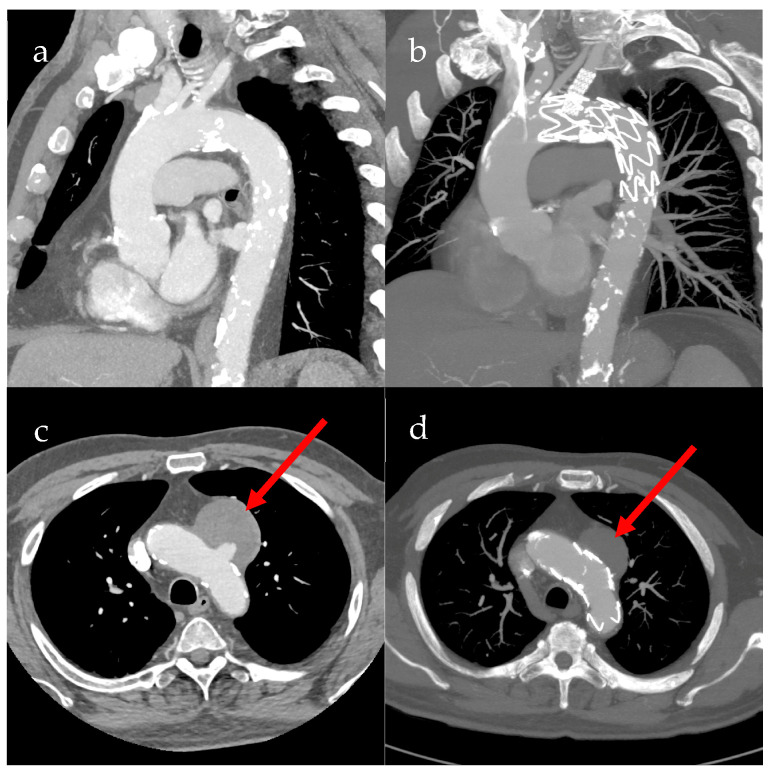
Pre- and postoperative CT scans of in situ fenestrated thoracic endovascular aortic repair (isfTEVAR). (**a**) Sagittal preoperative CT image of the aortic arch; (**b**) sagittal postoperative CT image of the aortic arch; (**c**) transverse preoperative CT image of the aortic arch, red arrow showing perfused penetrating aortic ulcer (PAU); (**d**) transverse postoperative CT image of the aortic arch, with red arrow showing non-perfused PAU.

Alternatively, in urgent treatment indications, isfTEVAR is a method that provides an off-label, off-the-shelf solution. The penetration of the thoracic stent graft can either be wire- or laser-assisted. Following the fenestration, the covered bridging stent is implanted. The goal is a treatment of pathologies regarding Zone 3. Pre- and postoperative CT scans of a patient with penetrating aortic ulcer after treatment with isfTEVAR are shown in Figure 1 [3].

**Figure 2:**
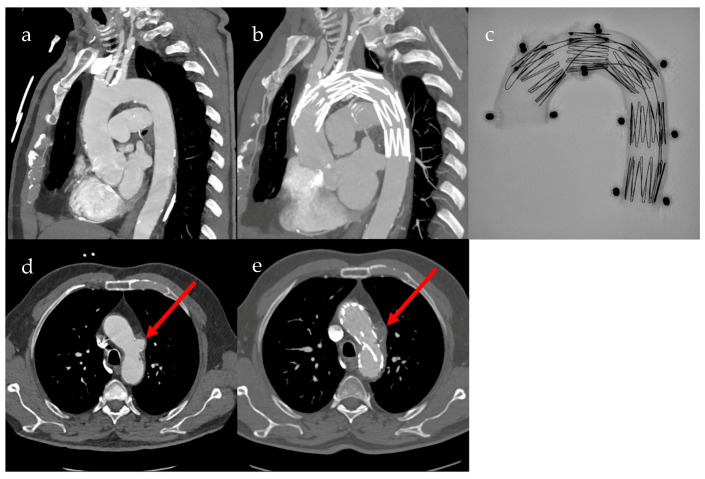
Pre- and postoperative CT scans of custom-made fenestrated thoracic endovascular aortic repair (cmfTEVAR) using the Najuta stent graft. (**a**) Sagittal preoperative CT image of the aortic arch; (**b**) sagittal postoperative CT image of the aortic arch; (**c**) X-ray image of the Najuta stent graft in aortic arch model; (**d**) transverse preoperative CT image of the aortic arch, with red arrow showing perfused penetrating aortic ulcer (PAU); (**e**) transverse postoperative CT image of the aortic arch, with red arrow showing non-perfused PAU.

Fenestrated grafts are specialized custom-made fenestrated stent grafts (cmfTEVAR) for the aortic arch to address anatomical challenges associated with standard thoracic stent grafts and extra-anatomic debranching of supra-aortic vessels. Despite technological advancements, aortic arch repairs involving the manipulation of supra-aortic vessels remain challenging and carry the inherent risks of major complications, such as stroke and death. Therefore, the Najuta (Kawasumi Laboratories, Inc., Tokyo, Japan) fenestrated thoracic stent graft, a custom-made solution, was designed to achieve a proximal landing between Zones 0 and 2. This design aims to preserve antegrade flow in supra-aortic vessels, eliminating the need for additional maneuvers in target arteries or the deployment of adjunctive components. The Najuta device represents a bespoke stent graft designed with singular or multiple unsupported fenestrations strategically positioned along its greater curvature to effectively maintain the blood supply through the arch vessels. Pre- and postoperative CT scans of a patient with penetrating aortic ulcer after treatment with cmfTEVAR, as well as an X-ray image of the Najuta fenestrated custom-made stent graft in aortic arch model, are shown in Figure 2 [4]. 

The branched custom-made grafts (cmbTEVAR) are engineered to achieve a proximal landing in the ascending aorta, just distal to the coronary arteries, as it is also the case with cmfTEVAR, namely from Zone 0 to Zone 2. Before a treatment using cmbTEVAR, a debranching of one or two supra-aortic vessels is needed. There are cmbTEVAR with one or two branches available. The delivery system comes precurved, allowing it to conform to the curvature of the aortic arch during advancement and ensuring self-alignment of the funnels with the target vessels. At the distal end, the graft can either provide a seal in the proximal descending aorta or be extended further distally in conjunction with other thoracic stent grafts [5].

None of the devices and endovascular techniques are fit for all anatomies and patients. In emergency settings, it is not possible to treat the patient with custom-made devices. Therefore, the treatment concept of every patient is individual.

The goal of this study is to capture and evaluate the clinical and technical success of endovascular therapy of the aortic arch and thoracic aorta and compare the various endovascular treatment options. The primary focus is on intraoperative procedure-specific variables and short-term postoperative outcomes.

## 2. Materials and Methods

This retrospective study includes all patients between January 2010 and March 2024 that underwent an endovascular treatment of aortic arch and thoracic aortic pathologies without acceptable central landing zone for standard TEVAR. An acceptable landing zone was defined as a minimum of 2 cm for sufficient sealing. All patients were not suitable for open aortic arch repair. The surgeries were all conducted in a hybrid operating room at the University Hospital Düsseldorf. The Philips AlluraClarity system (Amsterdam, the Netherlands) was used as the X-ray imaging system, and the ACIST CVi (Eden Prairie, MN, USA) was employed as the contrast agent injector.

### 2.1. Inclusion and Exclusion Criteria

Included were all patients that received an endovascular treatment of thoracic aortic pathologies without acceptable central landing zone of the graft in aortic arch in the Department of Vascular and Endovascular Surgery at the University Hospital Düsseldorf between January 2010 and March 2024. Only patients older than 18 years were included, as well as elective procedures and emergencies.

### 2.2. Data Analysis

Data analysis was performed with IBM SPSS29 (IBM Corporation, Armonk, NY, USA). All patients were divided into groups depending on their treatment method, thereupon those groups were compared by using the most important pre-, intra- and postoperative variables. The data analysis process involved partitioning the gathered data into four distinct groups, delineated by the specific surgical procedures each group underwent. Subsequently, a comprehensive comparison was conducted utilizing the descriptive analysis function. Within this analytical framework, all pertinent metrics were assessed, with a particular focus on the mean values, accompanied by the corresponding minimum (min) and maximum (max) values. The inclusion of standard deviation (SD) provided additional insights into the variability within each dataset, contributing to a more nuanced understanding of the observed trends and disparities among the different surgical intervention groups. This meticulous examination aimed to elucidate not only the central tendencies but also the overall spread and dispersion of data points, offering a comprehensive overview of the dataset’s characteristics for each treatment category. This retrospective study was conducted in accordance with the Declaration of Helsinki. The protocol was approved by the Ethics Committee of the Faculty of Medicine of Heinrich Heine University, Düsseldorf (2022-1905_1).

## 3. Results

### 3.1. Patients’ Characteristics 

This study contains 21 patients who underwent an endovascular treatment of aortic arch and thoracic aortic pathologies without acceptable proximal landing zone for standard TEVAR. A total of 17 of 21 patients were male (81%), with a mean age of 70.9 ± 9 years. The patients had an average ASA score of 3. Two of 21 patients (9.5%) were emergencies and received a treatment by using the chimney technique. Table 1 summarizes the patients characteristics and comorbidities.

### 3.2. Outcome 

The most treated aortic pathologies were penetrating aortic ulcers (PAUs) and chronic post-dissection aneurysms. Of 21 patients, seven patients were treated with isfTEVAR, five received a treatment with the chTEVAR, six patients were treated with cmfTEVAR, and three patients were treated with cmbTEVAR. Table 2 summarizes the treatment methods, implanted grafts and diagnosis of the patients. 

The patients were compared based on key intraoperative and postoperative variables, including the duration of the operation, contrast agent administration, as well as fluoroscopy time and exposure duration. Furthermore, postoperative complications, such as the need for reintervention, endoleaks, pseudoaneurysms, spinal cord ischemia, limb ischemia, in-hospital mortality, postoperative dialysis, sepsis, stroke, myocardial infarction and postoperative retrograde aortic dissection, were compared among them.

The patients were treated in four different ways. The first group of patients was treated with the chimney technique (chTEVAR); five patients can be categorized as 80% male, with an average age of 63.4 ± 9.5 years and an average BMI of 30.8 ± 11.2 kg/m^2^. In three out of five cases GORE TAG thoracic aortic stent grafts were used. Two patients were treated with COOK Zenith Alpha aortic thoracic stent grafts. For supra-aortic vessels, Jotec E-Ventus covered stents were used in four cases, and a GORE Viabahn VBX covered stent was used in one case, with in a chimney graft technique. All patients were treated with one chimney. Four chimneys were constructed for the left subclavian artery and one for the left common carotid artery. The proximal landing zone for chTEVAR in four cases was Segment 2 and in one case Segment 1 of the aorta, based on Ishimaru zones. Table 3 summarizes the patients’ characteristics for each treatment method.

The second group of patients was treated with in situ fenestrated TEVAR and included seven patients, 86% male, with an average age of 71 ± 10.3 years and an average BMI of 27.8 ± 4.6 kg/m^2^. The in situ fenestration was performed with a laser in all cases. For all seven cases, a COOK Zenith Alpha thoracic stent graft was used. The most commonly used bridging covered stents to connect the newly formed fenestration to the target artery were GORE Viabahn VBX in four cases, a Bentley BeGraft stent graft in two cases and a Bentley BeGraft Plus in one case. The proximal landing zone for isfTEVAR was Segment 2 of the aorta based on Ishimaru zones. All fenestrations of the thoracic stent graft were performed for the left subclavian artery. 

The third group of patients was treated with custom-made fenestrated TEVAR; this group, consisting of six patients, can be categorized as 83.3% male, with an average age of 74.3 ± 4.8 years and an average BMI of 27.1 ± 3.9 kg/m^2^. For all patients in this group, the Najuta fenestrated stent graft was used. No debranching or bridging covered stents were needed for the supra-aortic vessels. All supra-aortic vessels were patent after the implantation of cmfTEVAR. The proximal landing zone for cmfTEVAR was Segment 0 of the aorta based on Ishimaru zones.

The fourth group of patients was treated with custom-made branched TEVAR; three patients can be categorized in this group as 66.7% male, with an average age of 76.3 ± 5 years and an average BMI of 27.1 ± 6.8 kg/m^2^. The custom-made branched stent graft used in one case was a Terumo Aortic custom-made stent graft and the Nexus Artivion in two other cases. The Terumo Aortic cmbTEVAR stent graft had two branches. The debranching of the supra-aortic arteries was 2 months before the cmbTEVAR procedure was performed. As a preparation procedure, these patients received a carotid–subclavian bypass in one case, for the patient treated with Terumo Aortic, and a carotid–carotid–subclavian bypass for the two cases treated with Nexus Artivion. The proximal landing zone for cmbTEVAR was Segment 0 of the aorta based on Ishimaru zones.

In the comparison of these groups, there are significant differences in many factors. Intraoperative variables such as the procedure time, the fluoroscopy time and the radiation dose, as well as the usage of a contrast agent are important. The procedure time in all the analyzed treatment methods was very different. The longest surgeries were made with cmbTEVAR and isfTEVAR. The average duration of a surgery made with cmbTEVAR was 312 ± 92 min, while the average duration of a surgery made by isfTEVAR took 265 ± 73 min. The treatment using chTEVAR was faster than both of the other treatments. The average duration of a surgery with chTEVAR was 182 ± 58 min. The shortest average operating time was achieved using cmfTEVAR. The average duration was 150 ± 29 min, which is almost half the duration as needed for cmbTEVAR or the isfTEVAR. Table 4 summarizes the average duration of the procedure for each treatment method.

Furthermore, there were also big differences in the fluoroscopy time, the radiation dose and the usage of contrast agent. The longest fluoroscopy times were in operations with isfTEVAR and cmbTEVAR. The mean duration of fluoroscopy in patients treated by isfTEVAR was 44:38 ± 11:11 mm:ss, while the mean duration of fluoroscopy in patients treated by cmbTEVAR was 47:37 ± 08:57 mm:ss. Patients who were treated with chTEVAR were exposed to a shorter fluoroscopy time, with an average of 21:59 ± 10:25 mm:ss. The shortest fluoroscopy time was recorded in the group of patients that received the treatment with cmfTEVAR. The mean fluoroscopy time in these groups was 18 ± 05:32 mm:ss. In conclusion, the results for the duration of an operation and the fluoroscopy time within these operations are comparable. This changes upon closer inspection on the usage of contrast agent. Treatments with chTEVAR have the highest usage of contrast agent, with an average of 79 ± 32 mL. Patients who received an operation with cmbTEVAR show a reduced usage of contrast agent, averaging 55 ± 18 mL. This was even lower in the group of patients that were treated with isfTEVAR. The treatments with this technique demonstrated an average usage of 47 ± 32 mL. Compared to these interventions, the approach with cmfTEVAR was again better, with an inferior usage of contrast agent of 40 ± 18 mL in average. 

There were no relevant differences in the radiation dose needed for the treatment. The mean radiation dose in the patients treated by chTEVAR was 181.6 ± 87.4 Gycm^2^, for isfTEVAR it was 110.7 ± 57.8 Gycm^2^, for cmfTEVAR it was 142.3 ± 108.9 Gycm^2^ and for cmbTEVAR it was 168.3 ± 55 Gycm^2^. Table 5 summarizes the fluoroscopy time, radiation dose and amount of contrast agent for each treatment method.

Thereupon, intra- and postoperative variables, such as technical success, the rate of reinterventions, endoleaks and the in-hospital deaths, and the postoperative complications such as retrograde aortic dissection, stroke, spinal cord ischemia, limb ischemia, sepsis, pseudoaneurysms, myocardial infarction and acute kidney insufficiency requiring dialysis were analyzed. Table 6 summarizes the complication rates of each treatment method.

The overall mortality rate was 9.5% (2/21), including emergencies. In elective settings, the in-hospital mortality was 5.2% (1/19). Both patients were treated with chTEVAR.

In the group of patients treated by chTEVAR, the rate of reinterventions was 20% (1/5). This patient presented to our emergency department as a critical case with sepsis and a rupture in the aortic arch following the implantation of a stent graft in the thoracic aorta by penetrating aortic ulcer one year before. This patient was treated with chTEVAR for the left common carotid artery. The reintervention was due to a complicated stent graft infection and aortic rupture at the distal end of the thoracic aortic stent graft. After the implantation of the second TEVAR, dialysis was needed. Despite the reintervention and the treatment of the sepsis, the patient passed away. The other patient who passed away after elective treatment with chTEVAR developed a postoperative type A dissection. Subsequently, she underwent cardiac surgery, during which she suffered an intracranial hemorrhage intraoperatively. This ultimately led to the patient’s death during hospitalization. 

The patients who received isfTEVAR needed no reintervention. The rate of technical success was 85.7% (6/7). The unsuccessful operation failed because the fenestration by laser made a hole lateral to the planned position, which led to the stent for the subclavian vessel being implanted outside the prothesis. Subsequently, the patient exhibited neurological deficits, indicating an underperfusion of the vertebral artery. A stroke was confirmed in the stroke CT scan, leading to the postoperative establishment of a carotid–subclavian bypass. The stroke rate includes one patient who experienced vision disturbances that improved over time.

The group of patients that were treated by using cmfTEVAR needed no reintervention. However, there was a pseudoaneurysm of the external iliac artery, so surgical repair was needed. The rate of technical success was 100% (6/6), and there were no other postoperative complications. 

The patients that received cmbTEVAR needed no reintervention. However, every patient treated by cmbTEVAR needed a debranching in a previous surgery. 

The overall stroke rate was 9.5% (2/21), with both strokes being found in the group of patients treated with isfTEVAR. The overall rate of myocardial infarction was 0% (0/21). Furthermore, postoperative limb ischemia and spinal cord ischemia were also compared among the different groups, and no differences were found. None of the treated patients exhibited postoperative limb or spinal cord ischemia. The covered lengths of the aorta with the thoracic aortic stent grafts for chTEVAR and isfTEVAR differed depending on the patient’s anatomy and the lengths of the stent grafts used, ranging between 109 mm and 165 mm. 

Nonetheless, distinct variations emerged in terms of the length of hospitalization among different treatment groups. The patients undergoing chTEVAR exhibited the lengthiest hospital stays, with an average duration of 41.4 ± 54.5 days. It is crucial to highlight that this group includes a patient with a complicated infection of stent grafts, significantly skewing the overall average. Subsequent to the chTEVAR cohort, the patients who receiving therapy with cmbTEVAR experienced an average hospital stay of 13.3 ± 3.2 days. This duration closely parallels that of patients treated with cmfTEVAR, who were hospitalized for an average of 12.5 ± 6.3 days. Table 7 summarizes the average hospital stay for each treatment method.

In contrast, the shortest hospital stays were observed in patients treated with isfTEVAR, where the average hospitalization duration was notably shorter, at 9.9 ± 2.3 days. These findings underscore the potential influence of the chosen treatment method on the overall duration of hospitalization, with notable variations dependent on the specific technique employed. 

## 4. Discussion

The results of this single-center study indicate that the endovascular treatment of the aortic arch and thoracic aortic pathologies with no acceptable landing zone for standard TEVAR procedure is safe, with acceptable results for patients who are not suitable for open surgery. Two patients died, who were treated with chTEVAR. We had no in-hospital mortality for the patients who were treated with isfTEVAR, cmfTEVAR and cmbTEVAR. In comparison to the endovascular therapy of the aortic arch, Thoralf et al. reported an overall mortality rate of 8.9% for open arch replacement, with a lower rate of 6% in elective procedures [6]. On the other hand, the mortality rate for endovascular treatment was 10.5%, with a specific rate of 5.2% for elective procedures [6]. Our mortality rate was similar to the reported results. None of the patients in our study were suitable for open aortic arch replacement.

The duration of surgical procedures bears substantial clinical significance and, as underscored by Cheng et al., demonstrates a correlation with an elevated vulnerability to infections [7]. It is imperative to minimize the operative time to mitigate surgery-induced infections and their associated complications. Notably, procedures involving cmfTEVAR emerge as particularly salient in this regard, exhibiting a mean operative duration of 150 min. The intraoperative results of isfTEVAR are comparable to the results of cmbTEVAR. It is important to note that all cases treated by cmbTEVAR received a debranching of the supra-aortic vessels 2 months before, which is not added to the time of the operation in this study and would increase the duration if added. 

In delving into a comparative analysis of operative durations, it is crucial to acknowledge the substantial influence wielded by the operating surgeon, chosen endovascular technique and complexity of aortic pathology. The variability in surgical durations underscores the intricate interplay of surgical skill, experience and procedural complexity, all of which contribute to the overall time spent in the operating room. Therefore, meticulous attention to surgical duration becomes pivotal not only for procedural efficiency but also for the mitigation of postoperative complications, aligning with the broader objective of optimizing patient outcomes in the realm of vascular interventions. 

In addition, the duration of fluoroscopy and the use of contrast agents are of great significance. Macariello and Mehran et al. describe that post-procedural contrast-induced nephropathy (CIN) occurred in 10–30% of patients [8,9]. The occurrence of CIN was particularly associated with the amount of contrast agent used; therefore, minimizing contrast agent usage is crucial for patient well-being. In our study, we did not analyze the incidence of postoperative CIN, but we investigated postoperative acute renal insufficiency requiring dialysis. However, as reported by Macariello and Mehran et al., one can anticipate a postoperative CIN incidence of 10–30%, dependent on the use of contrast agents and associated risk factors [8,9]. Our results suggest that the lowest contrast agent utilization is achieved, especially in treatments involving cmfTEVAR and isfTEVAR.

Additionally, perioperative and postoperative factors are crucial and influence the choice of surgical technique. Technical success and reinterventions, for example, are important considerations. We achieved a 100% technical success rate in operations utilizing chTEVAR; however, reinterventions were required in one out of five cases (20%). In terms of technical success, our results are comparable to other studies. Huang et al. reported a technical success rate of 84% [10], while Luo et al. achieved a 100% technical success rate, with reinterventions necessary in 0–0.5% of their patients [11]. Li et al. reported a reintervention rate of 4.1%. Additionally, the occurrence of strokes, especially in aortic arch surgeries, is a significant postoperative complication [12]. The results vary between 2.6% and 5.3% [2,12]. We observed one minor and one major stroke in the group of patients treated with isfTEVAR. 

Another commonly noted complication is the occurrence of endoleaks, with results varying between 9–18% [2,10,11,12,13]. We had no endoleaks during the in-hospital stay. Furthermore, Wang et al. reported a shorter operation duration of 129 ± 21 min for double chTEVAR, compared to our 182 ± 58 min. Our patients were treated with single chTEVAR. They also documented a radiation exposure time of 58 ± 19 min and a contrast agent usage of 173 ± 22 mL, which is significantly longer and higher than our recorded values of 22 ± 10 min for radiation exposure and 79 ± 32 mL for contrast agent usage [13]. 

Compared to chTEVAR, isfTEVAR demonstrates a superior complication profile. Li et al. reported an endoleak rate of 4.7% and a stroke rate of 3.4% [14]. The technical success rate is also notably high in existing literature. While Li et al. reported a technical success rate of 97.3%, a systematic review by Houérou et al. indicated a rate of 98% [14,15]. The latter reported an average endoleak rate of 8.3% and a stroke rate of 4.5% and additionally found a reintervention rate of 6% [15]. These values are in line regarding the rates for reinterventions but significantly lower than our observed rates for strokes (28.6%), which can be attributed, in part, to the size of our cohort. Conversely, our incidence of postoperative endoleaks was 0%. 

In our results, cmfTEVAR demonstrated the most favorable outcomes in both perioperative and postoperative factors. Similar findings are reflected in the existing literature. The technical success rate of the Najuta stent graft is notably high, ranging between 92.3% and 100%, as reported by Isernia, Iida, Toya, Sato and Fukushima, among others [4,16,17,18,19]. These figures align with our observed results. We had no postoperative strokes after cmfTEVAR. Stroke rates varied in the literature, with Toya et al. and Maeda et al. reporting rates between 0% and 5% [17,20], consistent with our findings. In contrast, Iida et al. and Sato et al. reported stroke rates ranging from 10% to 16.7% [16,18].

Additionally, endoleaks after cmfTEVAR are a significant postoperative complication in endovascular treatments. Discrepancies exist in the literature, with Toya et al. and Fukushima et al. reporting endoleak incidences of 0% and 7.7% after cmfTEVAR [17,19], respectively, matching our results. Conversely, Iida et al. and Sato et al. reported endoleaks in 25% and 27.8% of the treated patients [16,18]. Besides postoperative factors, perioperative outcomes are also intriguing to observe. The reported operation durations for cmfTEVAR in the selected literature range from 90 to 158 min on average [4,19], consistent with our average duration of 150 min. The average fluoroscopy time ranged between 21 and 25 min [4,19], slightly higher than our results. Similarly, contrast agent usage, a risk factor for contrast-induced nephropathy (CIN), was higher in Isernia et al. and Fukushima et al., with an average contrast agent usage of 120 mL and 194 mL [4,19], respectively, compared to our results.

The duration of hospital stay is another crucial perioperative factor, contingent on comorbidities and the economic reimbursement tied to patient length of stay. While interpretating our results for the length of hospital stay, it is crucial to bear in mind that there can be systematical differences between hospitals and countries. In Germany, it is very important to know the home care situation of the patient. If it is uncertain, or if the patients cannot take care of themselves, they must be provided with appropriate care. Often, this social service must be organized by hospital staff, leading to patients spending longer periods in the hospital. While Fukushima et al. reported a hospital stay of 7 days [19], Sato et al. reported an average stay of 19.5 days [18]. Our results, with an average stay of 12.5 days, fall between these two extremes. In addition to these factors, applicability is a critical consideration. Hauck et al. investigated applicability, revealing that the Najuta stent graft exhibited the highest applicability compared to other endovascular stent grafts for treating residual type A dissections after ascending aortic replacement. The Najuta stent graft was usable in 82.2% of the patients (83 out of 101) included in the study [21]. 

As a final treatment option, our study found that cmbTEVAR, in terms of perioperative factors, yielded inferior results compared to isfTEVAR or cmfTEVAR. Considering the postoperative factors, the results indicate that the rate of complications is lowest here. Studies indicate a technical success rate ranging from 93% to 100% [22,23], aligning with our findings. This consistency extends to the operation duration and fluoroscopy time. In the selected studies, the average operation duration varied between 220 min and 286 min [5,22,23], with our average of 312 min slightly exceeding this range. It is important to mention that this is only the time needed for cmbTEVAR, but all our patients received a debranching of the supra-aortic vessels 2 months before as preparation for the cmbTEVAR, which is not included in the operation duration. The average fluoroscopy time ranged between 47 min and 74 min [5,22], aligning with our recorded values. However, this correlation does not extend to the usage of contrast agents. Verhoeven et al. reported an average contrast agent usage of 277 mL [22], significantly higher than our results. Conversely, the average length of hospital stay is comparable. In the selected literature, it is reported to range between 8 and 14 days [5,22,23], overlapping with our observed results.

In this context, it is interesting to note that no retrograde Type A dissection was observed in the group of patients treated with cmfTEVAR and cmbTEVAR. This is noteworthy because one might expect that the risk for this complication would be higher with a more proximal landing, as was the case in these treatments. Consequently, these treatment options might offer a significant advantage over a hybrid surgical approach, as the risk for a bird-beak formation, and consequently the risk for a Type 1a endoleak, could be lower.

This study has significant limitations, primarily because comparing outcomes regarding complications is not necessarily feasible due to the diverse indications and diagnoses under treatment. However, this does not apply to intraoperative key factors such as operation duration, contrast agent usage and radiation exposure, as these are independent from the diagnosis.

The results are noteworthy; however, it is essential to bear in mind that the group of analyzed patients in this study is small and accordingly, the statistical analysis may not be very conclusive. For instance, the overrepresentation of complications in certain groups compared to other studies with a larger patient sample size demonstrates this. In addition, these techniques are still relatively new, and their success heavily depends on the skill of the surgeon. It can be assumed that with an increasing number of cases and surgical experience, the complication rate will decrease. It is also important to note that we have compared four methods, which have a different approach, and that revascularization starting from Zone 0 can be more challenging than the endovascular treatment starting from Zone 2. Nevertheless, this work provides a good overview of different endovascular treatment methods and the outcomes for high-risk patients, who are unsuitable for open surgery due to aortic arch and thoracic aortic pathologies, with no acceptable landing zone for standard TEVAR.

## 5. Conclusions

Complex endovascular procedures of the aortic arch with custom-made or surgeon-modified aortic stent grafts offer a safe solution, with acceptable complication rates for high-risk patients who are not suitable for open aortic arch replacement. In terms of procedure-related parameters and complication rates, the custom-made fenestrated TEVAR is potentially advantageous compared to chimney TEVAR, in situ fenestrated TEVAR and custom-made branched TEVAR.

## Figures and Tables

**Table 1:** Patients’ characteristics. Data are presented in absolute numbers and percentage shares.

Variables	Number of Patients (%)
Age (years)	70.9 ± 9
Sex (male)	17 (81.0)
Medical history	
Hypertension	18 (85.7)
Diabetes	5 (23.8)
Chronic kidney insufficiency requiring dialysis	1 (4.8)
Ischemic heart disease	4 (19.0)
COPD	4 (19.0)
Cerebral vascular disease	2 (9.5)
Dyslipoproteinemia	6 (28.6)
Nicotine abuse	9 (42.9)
ASA score	
2	2 (9.5)
3	15 (71.4)
4	4 (19.0)

**Table 2:** List of enclosed patients includes the utilized stent grafts, diagnosis and baseline data.

N	Treatment	Emergency	Age	Sex	BMI	ASA	Diagnosis	Stent Grafts
1	chTEVAR	Yes	63	M	16.6	4	Aortic arch rupture post graft infection	GORE TAG + GORE Viabahn VBX
2	chTEVAR	Yes	69	M	34	4	PAU	COOK ZTA + JOTEC E-Ventus
3	chTEVAR	No	47	M	54.9	3	Thrombus of descending aorta	COOK ZTA + JOTEC E-Ventus
4	chTEVAR	No	70	M	25.4	3	Chronic post-dissection aneurysms	GORE TAG + JOTEC E-Ventus
5	chTEVAR	No	68	F	31.2	3	Chronic post-dissection aneurysms	GORE TAG + JOTEC E-Ventus
6	isfTEVAR	No	81	M	35.6	4	PAU	COOK ZTA + GORE Viabahn VBX
7	isfTEVAR	No	79	F	23	3	PAU	COOK ZTA + Bentley BeGraft
8	isfTEVAR	No	72	M	23.4	2	Arcus aortae duplex	COOK ZTA + Bentley BeGraft
9	isfTEVAR	No	63	M	28.4	4	PAU	COOK ZTA + GORE Viabahn VBX
10	isfTEVAR	No	55	M	29.1	3	PAU	COOK ZTA + GORE Viabahn VBX
11	isfTEVAR	No	65	M	23.7	3	PAU	COOK ZTA + Bentley BeGraft Plus
12	isfTEVAR	No	83	M	29.8	3	PAU	COOK ZTEG + GORE Viabahn VBX
13	cmfTEVAR	No	82	M	32.8	3	Chronic post-dissection aneurysms	Kawasumi Najuta
14	cmfTEVAR	No	73	M	26.9	3	Chronic post-dissection aneurysms	Kawasumi Najuta
15	cmfTEVAR	No	71	M	23.9	3	Chronic post-dissection aneurysms	Kawasumi Najuta
16	cmfTEVAR	No	69	M	25.8	3	Chronic post-dissection aneurysms	Kawasumi Najuta
17	cmfTEVAR	No	74	F	30.5	3	PAU	Kawasumi Najuta
18	cmfTEVAR	No	76	M	22.7	2	PAU	Kawasumi Najuta
19	cmbTEVAR	No	81	M	30.3	3	Aneurysm	Terumo Aortic
20	cmbTEVAR	No	71	M	31.7	3	PAU	Nexus Artivion
21	cmbTEVAR	No	77	F	19.2	3	Type 1a endoleak	Nexus Artivion

chTEVAR, chimney thoracic endovascular aortic repair. cmbTEVAR, custom-made branched thoracic endovascular aortic repair. cmfTEVAR, custom-made fenestrated thoracic endovascular repair. isfTEVAR, in situ fenestrated thoracic endovascular aortic repair. F, female. M, male. PAU, penetrating aortic ulcer.

**Table 3:** Patients’ characteristics for each treatment method.

Treatment	N (%)	Male-Sex (%)	Age (Years)	BMI ± SD (kg/m^2^)
chTEVAR	5 (23.8)	4 (80)	63.4 ± 9.5	30.8 ± 11.2
isfTEVAR	7 (33.3)	6 (86)	71 ± 10.3	27.8 ± 4.6
cmfTEVAR	6 (28.6)	5 (83)	74.3 ± 4.8	27.1 ± 3.9
cmbTEVAR	3 (14.3)	2 (67)	76.3 ± 5	27.1 ± 6.8

chTEVAR, chimney thoracic endovascular aortic repair. cmbTEVAR, custom-made branched thoracic endovascular aortic repair. cmfTEVAR, custom-made fenestrated thoracic endovascular repair. isfTEVAR, in situ fenestrated thoracic endovascular aortic repair.

**Table 4:** Average duration of procedure for each treatment method in minutes.

Treatment	Min (min)	Max (min)	Average ± SD (min)
chTEVAR	109	266	182 ± 58
isfTEVAR	165	344	265 ± 73
cmfTEVAR	105	195	150 ± 29
cmbTEVAR	207	377	312 ± 92

chTEVAR, chimney thoracic endovascular aortic repair. cmbTEVAR, custom-made branched thoracic endovascular aortic repair. cmfTEVAR, custom-made fenestrated thoracic endovascular repair. isfTEVAR, in situ fenestrated thoracic endovascular aortic repair.

**Table 5:** Fluoroscopy time, radiation dose and amount of contrast agent for each treatment.

Treatment		Min	Max	Average ± SD
chTEVAR	Fluoroscopy time (mm:ss)	11:52	36:06	21:59 ± 10:25
	Radiation dose (Gycm^2^)	74.2	310	181.6 ± 87.4
	Contrast agent (mL)	35	104	79 ± 32
isfTEVAR	Fluoroscopy time (mm:ss)	30:53	59:37	44:38 ± 11:11
	Radiation dose (Gycm^2^)	36.7	194.0	110.7 ± 57.8
	Contrast agent (mL)	26	117	47 ± 32
cmfTEVAR	Fluoroscopy time (mm:ss)	10:57	25:53	18:00 ± 05:32
	Radiation dose (Gycm^2^)	73.6	333	142.3 ± 108.9
	Contrast agent (mL)	22	68	40 ± 18
cmbTEVAR	Fluoroscopy time (mm:ss)	38:52	56:47	47:37 ± 08:57
	Radiation dose (Gycm^2^)	105	205	168.3 ± 55
	Contrast agent (mL)	40	75	55 ± 18

chTEVAR, chimney thoracic endovascular aortic repair. cmbTEVAR, custom-made branched thoracic endovascular aortic repair. cmfTEVAR, custom-made fenestrated thoracic endovascular repair. isfTEVAR, in situ fenestrated thoracic endovascular aortic repair.

**Table 6:** Complication rates of each treatment method.

	chTEVAR % (N)	isfTEVAR % (N)	cmfTEVAR % (N)	cmbTEVAR % (N)
Technical success	100 (5/5)	86 (6/7)	100 (6/6)	100 (3/3)
Endoleak	0 (0/5)	0 (0/7)	0 (0/6)	0 (0/3)
Reintervention	20 (1/5)	0 (0/7)	0 (0/6)	0 (0/3)
Stroke	0 (0/5)	27 (2/7)	0 (0/6)	0 (0/3)
major	0 (0/5)	14 (1/7)	0 (0/6)	0 (0/3)
minor	0 (0/5)	14 (1/7)	0 (0/6)	0 (0/3)
Myocardial infarction	0 (0/5)	0 (0/7)	0 (0/6)	0 (0/3)
Limb ischemia	0 (0/5)	0 (0/7)	0 (0/6)	0 (0/3)
Pseudoaneurysm	0 (0/5)	0 (0/7)	17 (1/6)	0 (0/3)
Acute kidney insufficiency requiring dialysis	20 (1/5)	0 (0/7)	0 (0/6)	0 (0/3)
Sepsis	20 (1/5)	0 (0/7)	0 (0/6)	0 (0/3)
Death	40 (2/5)	0 (0/7)	0 (0/6)	0 (0/3)

chTEVAR, chimney thoracic endovascular aortic repair. cmbTEVAR, custom-made branched thoracic endovascular aortic repair. cmfTEVAR, custom-made fenestrated thoracic endovascular repair. isfTEVAR, in situ fenestrated thoracic endovascular aortic repair.

**Table 7:** Average hospital stays for each treatment method.

Treatment	Min (Days)	Max (Days)	Average ± SD (Days)
chTEVAR	3	137	41.4 ± 54.5
isfTEVAR	8	14	9.9 ± 2.3
cmfTEVAR	6	23	12.5 ± 6.3
cmbTEVAR	11	17	13.3 ± 3.2

chTEVAR, chimney thoracic endovascular aortic repair. cmbTEVAR, custom-made branched thoracic endovascular aortic repair. cmfTEVAR, custom-made fenestrated thoracic endovascular repair. isfTEVAR, in situ fenestrated thoracic endovascular aortic repair.

## Data Availability

The data presented in this study cannot be shared due to the privacy of individuals that participated in this study and ethical reasons. On justified interest, the data will be available from the corresponding author after approval from the responsible Ethical Committee.
